# Tranexamic Acid in Lower Extremity Endoprosthetic Reconstruction for Oncologic Indications: A Retrospective Comparative Study of 617 Patients

**DOI:** 10.1002/jso.70250

**Published:** 2026-04-15

**Authors:** Stephen W. Chenard, Akhil Rekulapelli, Riley S. Gilbertson, Connor S. Charton, William F. Hefley, Michael J. Colello, Jennifer L. Halpern, Herbert S. Schwartz, Daniel J. Johnson, Jonathan G. Schoenecker, Joshua M. Lawrenz

**Affiliations:** ^1^ Vanderbilt University School of Medicine Nashville TN USA; ^2^ Center for Bone Biology Vanderbilt University Medical Center Nashville TN USA; ^3^ Program in Cancer Biology Vanderbilt University Nashville TN USA; ^4^ Department of Orthopaedic Surgery Vanderbilt University Medical Center Nashville TN USA; ^5^ Department of Pathology, Microbiology, and Immunology Vanderbilt University Nashville TN USA; ^6^ Department of Pharmacology Vanderbilt University Nashville TN USA; ^7^ Monroe Carell Jr. Children's Hospital at Vanderbilt Nashville TN USA; ^8^ Department of Pediatrics Vanderbilt University Medical Center Nashville TN USA

**Keywords:** blood transfusion, limb salvage, pulmonary embolism, sarcoma, venous thromboembolism, venous thrombosis

## Abstract

**Background and Objectives:**

While tranexamic acid (TXA) reduces blood loss in orthopedic surgery, thromboembolic concerns in cancer patients have limited adoption in orthopedic oncology. This study evaluated TXA efficacy and safety in patients undergoing endoprosthetic reconstruction for oncologic indications.

**Methods:**

This retrospective single‐center study included 617 patients who underwent lower extremity endoprosthetic reconstruction for oncologic indications between 2000 and 2024. Patients were stratified by perioperative TXA administration (*n* = 166) versus no TXA (*n* = 451). The primary outcome was perioperative blood loss calculated using the Mercuriali method. Secondary outcomes included perioperative packed red blood cells (pRBC) transfusion, hospital length of stay, and 90‐day venous thromboembolic (VTE) complications.

**Results:**

TXA was associated with a 429 mL reduction in calculated perioperative blood loss (1878 ± 1168 mL vs. 2307 ± 1442 mL; *p* = 0.003). TXA was not associated with reduced intraoperative pRBC transfusion rates (31% vs. 33%; RR 0.96 [95% CI: 0.74–1.25], *p* = 0.752) but was associated with significantly reduced postoperative transfusion requirements (17% vs. 30%; RR 0.56 [95% CI: 0.39–0.81], *p* = 0.003). No significant differences existed in 90‐day VTE complications, reoperation rates, or mortality.

**Conclusions:**

Perioperative TXA use was associated with reduced blood loss and postoperative transfusion requirements without a detectable increase in thromboembolic complications, supporting TXA as a beneficial adjunct in musculoskeletal oncology limb salvage procedures.

**Abbreviations**
DVT: deep vein thrombosis
EBL: estimated blood loss
EBV: estimated blood volume
Hct: hematocrit
MI: myocardial infarction
PE: pulmonary embolism
POD: postoperative day
pRBC: packed red blood cells
TIA: transient ischemic attack
TXA: tranexamic acid

## 1 Introduction

Tranexamic acid (TXA) is an antifibrinolytic drug that was first widely used in obstetrics and trauma to reduce mortality caused by massive hemorrhage [1, 2, 3]. It has since been widely adopted by orthopedic surgeons in a variety of subspecialties including sports medicine [4, 5, 6], spine [7, 8, 9, 10, 11, 12], and arthroplasty [13, 14, 15, 16, 17, 18]. In these settings, TXA reduces blood loss, transfusion rate, and hospital length of stay, with robust evidence that the use of TXA does not increase the risk of deep vein thrombosis (DVT), pulmonary embolism (PE), or other venous thromboembolic (VTE) complications.

Despite its clinical utility, concerns remain regarding the use of TXA in patients with cancer [19, 20]. Patients with cancer are known to live with a hypercoagulable state, which predisposes them to forming blood clots, a phenomenon known as cancer‐associated thrombosis [21, 22, 23]. Patients with cancer have a ninefold increased risk of suffering a VTE compared to the general population [24]. Furthermore, VTE is the second leading cause of death among patients with cancer, surpassed only by the malignancy itself [25, 26]. Given the antifibrinolytic mechanism of TXA, there is theoretical concern that its use may further exacerbate thromboembolic complications in patients with cancer who are hypercoagulable at baseline.

As a result, adoption of TXA within orthopedic oncology has lagged behind other orthopedic subspecialties. Previous studies exploring the use of TXA in patients undergoing surgery for musculoskeletal malignancies have suggested that TXA is safe and effective, demonstrating reductions in blood loss and transfusion requirements without clear increases in thromboembolic complications [27, 28, 29, 30, 31, 32, 33, 34, 35, 36]. However, these early studies have frequently been limited by small sample sizes, heterogeneous tumor types, and variable surgical procedures. Furthermore, there is at least one contradicting report suggesting the use of TXA increases the risk of PE in patients with sarcoma [37], further contributing to ongoing controversy and uncertainty regarding the safety of TXA use in this population.

Given the substantial blood loss associated with large oncologic resections and limb salvage procedures, clarifying the safety and efficacy of TXA in orthopedic oncology is clinically important. Therefore, the purpose of this study was to evaluate the association between perioperative TXA administration, blood loss, and thromboembolic complications in a large cohort of patients undergoing lower extremity endoprosthetic reconstruction for oncologic indications.

## 2 Materials and Methods

This retrospective study was approved by the Institutional Review Board (IRB# 222300), with a waiver of informed consent. An institutional database of orthopedic oncology patients was used to identify all patients who underwent endoprosthetic reconstruction at a single tertiary academic hospital between January 1, 2000, and June 30, 2024.

Patients were included in this study if they underwent lower extremity endoprosthetic reconstruction for an oncological indication at the primary study institution in the time period indicated above. Oncologic indications were defined as primary bone sarcoma, metastatic carcinoma to bone, or other oncologic indication (including primary or metastatic soft tissue sarcoma with involvement of bone, multiple myeloma/plasmacytoma, lymphoma/leukemia, benign aggressive bone tumor, and metastatic bone sarcoma to bone where the endoprosthesis was for the metastatic lesion). Patients were excluded if they had a nononcologic indication for reconstruction (such as trauma or revision arthroplasty), were undergoing revision of a prior endoprosthetic reconstruction, had their endoprosthetic reconstruction performed at an outside institution, or lacked adequate perioperative anesthesia or medication records.

After applying these inclusion and exclusion criteria, a total of 617 patients were included in this study. A comprehensive retrospective review of patient electronic health records was conducted. Demographic, surgical, and outcome variables were extracted and stored in a HIPAA (Health Insurance Portability and Accountability Act)‐compliant REDCap database hosted on an encrypted server at the primary institution.

**Figure 1:**
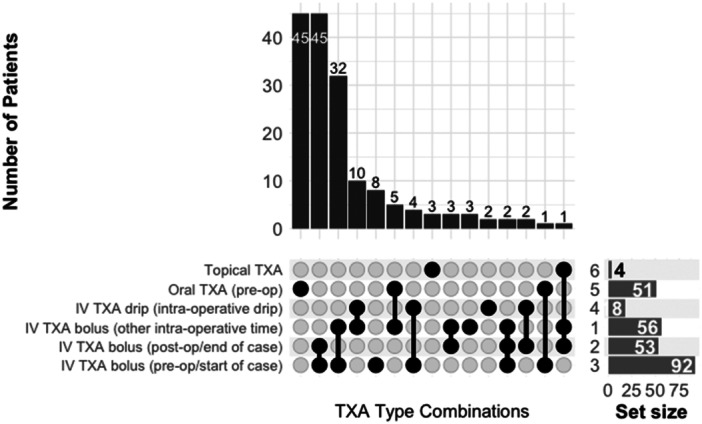
Upset plot demonstrating the most common routes and combinations of administration of tranexamic acid (TXA).

The study cohort was subdivided into two groups based on TXA administration. Patients were classified as TXA‐treated if they received TXA by any route (oral, intravenous, and/or topical) within the immediate perioperative period. Of the 166 patients who received TXA in this study, 27% received preoperative oral TXA alone (45/166), 27% received preoperative and postoperative IV boluses of TXA (45/166), and 19% received preoperative and intraoperative IV boluses of TXA (32/166). The remaining 27% of patients (44/166) received TXA by less common routes and combinations (Figure 1).

Baseline patient characteristics and surgical factors were similar between patients who received TXA (*n* = 166) and who did not receive TXA (*n* = 451) (Table 1). There were no detectable differences in age, sex, or BMI between treatment groups (all *p *> 0.05), although there were significantly fewer patients who self‐identified as current smokers in the group that received TXA (8% [13/166] vs. 15% [66/451]; *p* = 0.029). There were no detectable differences in major preoperative medical comorbidities between groups, including history of bleeding or thromboembolic conditions. There were no differences in preoperative use of aspirin or other antiplatelet agents between groups, however a significantly higher proportion of patients who received TXA had a direct factor Xa inhibitor such as apixaban or rivaroxaban listed in their preoperative home medications (5.4% [9/166] vs. 1.3% [6/451]; *p* = 0.007). This was matched by a lower proportion of TXA‐treated patients having a vitamin K antagonist like warfarin listed in their preoperative home medications compared to patients that did not receive TXA (1.2% [2/166] vs. 5.8% [26/451]; *p* = 0.016). There were no detectable differences in any oncologic or surgical factors, including presence of preoperative metastatic disease, indication for endoprosthetic reconstruction, or implant location or size (all *p* > 0.05).

**Table 1:** Demographic, medical, and surgical characteristics of study cohort.

	All patients (*n* = 617)	TXA (*n* = 166)	No TXA (*n* = 451)	*p*‐value^a^
Demographic information				
Age at surgery; years, mean (SD)	52.4 (20.9)	53.8 (21.6)	51.9 (20.6)	0.134
Female sex	50% (309)	54% (89)	49% (220)	0.287
BMI; kg/m^2, mean (SD)	28.0 (7.1)	27.8 (7.5)	28.1 (6.9)	0.578
Current tobacco use	13% (79)	8% (13)	15% (66)	**0.029**
Preoperative comorbidities				
Bleeding or clotting disorder	0.8% (5)	1.2% (2)	0.7% (3)	0.615
Deep vein thrombosis	8.1% (50)	7.8% (13)	8.2% (37)	0.880
Pulmonary embolism	2.8% (17)	2.4% (4)	2.9% (13)	0.999
Myocardial infarction	6.0% (37)	7.2% (12)	5.5% (25)	0.434
Transient ischemic attack/stroke	3.1% (19)	3.0% (5)	3.1% (14)	0.953
Diabetes	14% (85)	10% (17)	15% (68)	0.122
Chronic kidney disease	6.0% (37)	9.0% (15)	4.9% (22)	0.054
Preoperative home medications				
Vitamin K antagonist	4.5% (28)	1.2% (2)	5.8% (26)	**0.016**
Direct factor Xa inhibitor	2.5% (15)	5.4% (9)	1.3% (6)	**0.007**
Aspirin	15% (92)	17% (29)	14% (63)	0.284
Non‐aspirin antiplatelet agent	1.9% (12)	3.0% (5)	1.6% (7)	0.321
Heparin	9.1% (56)	7.2% (12)	9.8% (44)	0.329
Oncologic factors				
Known metastatic disease	57% (353)	58% (97)	57% (256)	0.710
Neoadjuvant chemotherapy	41% (255)	42% (70)	41% (185)	0.797
Neoadjuvant radiation	21% (131)	19% (31)	22% (100)	0.346
Surgical factors				
Indication for surgery				0.623
Primary bone sarcoma	36.5% (225)	31.9% (53)	38.1% (172)	
Metastatic carcinoma	34.2% (211)	36.7% (61)	33.3% (150)	
Other oncologic indication	29.3% (181)	31.3% (52)	28.6% (129)	
Implant anatomic location				0.748
Proximal femur	57% (350)	53% (88)	58% (262)	
Distal femur	32% (195)	34% (57)	31% (138)	
Proximal tibia	9.2% (57)	9.6% (41)	9.1% (41)	
Total femur	1.8% (11)	2.4% (4)	1.6% (7)	
Combined femur/tibia	0.6% (4)	0.6% (1)	0.7% (3)	
Resection length; cm, mean (SD)	146 (77)	148 (68)	146 (80)	0.868
Operative duration; min, mean (SD)	217 (82)	222 (75)	215 (84)	0.203

*Note:* Bolded values indicate statistical significance at a threshold of *p* < 0.05.

Abbreviations: BMI, body mass index; SD, standard deviation; TXA, tranexamic acid.

^a^ *p*‐value compares patients who did versus did not receive TXA. Categorical variables were compared using Fisher's exact test, and continuous variables were compared using the Mann–Whitney *U*‐test. Data presented as % (*n*), unless otherwise indicated.

The primary outcome of interest was total perioperative blood loss calculated using the method described by Mercuriali et al. (Formula 1) [38]. This is a well‐validated modification of the method initially proposed by Gross et al. [39], which uses total estimated blood volume (EBV), plus pre‐ and minimum postoperative hematocrit (Hct) values to estimate perioperative blood loss. The Mercuriali method is an improvement over the Gross method because it additionally accounts for perioperative blood transfusion volume, which is a known confounder of postoperative Hct values [40]. Each unit of transfused packed red blood cells (pRBC) was assumed to be 200 mL based on institutional practice, which is also consistent with prior literature [40].

Formula1:EstimatedPerioperativeBloodLoss(Mercurialietal),

$\mathrm{Perioperative lood Loss}\;\left(\mathrm{mL}\right)=\mathrm{Total Estimated Blood Volume}\times \;\left({\mathrm{Hct}}_{\mathrm{pre}}\right)\left({\mathrm{Hct}}_{\mathrm{pre}}-\;{\mathrm{Hct}}_{\mathrm{post}}\right)+\mathrm{transfused RBC volume}.$

Total EBV was calculated using the method proposed by Nadler et al. (Formula 2) [41]. This is a widely used method that incorporates patient sex, height, and weight to predict EBV:

Formula2:EstimatedBloodVolume(EBV)(Nadleretal),

$\mathrm{Male EBV}\;\left(l\right)=0.3669\times {\left(\mathrm{height},m\right)}^{3}+0.03219\times (\mathrm{weight},\mathrm{kg})+0.6041,$

$\mathrm{Female EBV}\;(l)=0.3561\times {\left(\mathrm{height},m\right)}^{3}+0.03308\times (\mathrm{weight},\mathrm{kg})+0.1833.$

Previous studies have reported total perioperative blood loss ranging from 1873 to 2461 mL for patients undergoing lower extremity endoprosthetic reconstruction without administration of TXA [29, 31]. Based on this prior literature, a sensitivity analysis was performed for this study using 2000 mL as an estimate of total perioperative blood loss. With 166 TXA‐treated and 451 untreated patients, two‐sided *α* = 0.05, and *β* = 0.2, this study had 80% power to detect a mean difference of approximately 317 mL of total perioperative blood loss between groups.

Secondary outcomes related to TXA efficacy included the proportion of patients that required an intraoperative or postoperative transfusion of pRBC, as well as the number of units of pRBC transfused perioperatively. The Surgeon reported estimated blood loss (EBL) was also extracted from operative reports and anesthesia care records. Length of postoperative hospital stay was calculated as the difference between the date of the index surgery and the date of discharge to any nonhospital location. Postoperative drain output volumes were not routinely or consistently documented, so this variable was not included.

Secondary outcomes related to TXA safety included 90‐day VTE complications, reoperation rates, and death. VTE complications included DVT, PE, myocardial infarction (MI), ischemic stroke/transient ischemic attack (TIA), or other VTE diagnosed by imaging. Reoperation was defined as any unplanned return to the operating room within 90 days. Death was defined as all‐cause mortality within 90 days postoperatively. All patients had at least 90 days of follow‐up or suffered a secondary safety outcome of interest within 90 days of their index surgery.

Secondary safety outcomes were assessed for the first 90 days postoperatively, as this is the time frame that the vast majority of surgery‐related VTE complications occur within [42]. This is a more stringent cut‐off than the 30‐day or 6‐week minimum follow‐up used in previous similar studies examining the risks of TXA in endoprosthetic reconstruction [28, 29], while maintaining a larger sample size than if minimum follow‐up were to be increased to 1 or 2 years. Given the high baseline risk of mortality in the orthopedic oncology patient population, this is a biologically relevant duration to answer the secondary study question, without unnecessarily sacrificing statistical power.

Aside from routine postoperative physical examinations, asymptomatic patients were not systematically screened for thromboembolic complications during the study period. Patients presenting postoperatively with symptoms suggestive of DVT underwent formal diagnostic evaluation with venous duplex ultrasonography. Those with symptoms concerning PE were assessed using computed tomography pulmonary angiography or ventilation‐perfusion scanning. Patients were only considered to have suffered a thromboembolic complication if the event was confirmed by objective imaging or diagnostic studies.

Statistical analyses were performed in R (Version 4.5.2; R Foundation for Statistical Computing, Vienna, Austria). When comparing features between treatment groups, categorical variables were compared using Fisher's exact test, and continuous variables were compared using the Mann–Whitney *U*‐test. Firth's penalized univariable logistic regression was used to identify predictors of thromboembolic events. Statistically valid multivariable logistic regression was not possible due to a limited number of outcomes of interest. All tests were two‐sided, and the threshold for statistical significance was set at *p* < 0.05.

## 3 Results

### 3.1 Blood Loss

Mean perioperative blood loss calculated using the Mercuriali method (Formula 1) was 1878 ± 1168 mL in the TXA group versus 2307 ± 1442 mL in the no‐TXA group (Table 2). TXA administration was associated with a mean reduction in calculated perioperative blood loss of 429 mL (95% CI: 207–651 mL; *p* = 0.003), which was larger than the minimum difference of 317 mL that this study was powered to detect. Mean surgeon‐reported intraoperative EBL was 427 ± 311 mL in the TXA group compared with 588 ± 545 mL in the no‐TXA group. Administration of TXA was associated with a mean reduction in surgeon‐reported EBL of 161 mL (95% CI: 92–230 mL; *p* < 0.001) (Table 2).

**Table 2:** Reduction in blood loss and units of packed red blood cell transfused perioperatively.

	TXA (*n* = 166)	No TXA (*n* = 451)	Mean reduction (95% CI)	*p*‐value^a^
Blood loss (mL)				
Calculated perioperative blood loss (Mercuriali)	1878 (1168)	2307 (1442)	429 (207–651) mL	**0.003**
The surgeon reported intraoperative blood loss	427 (311)	588 (545)	161 (92–230) mL	**< 0.001**
Transfusion volumes (units pRBC)				
Intraoperative	1.9 (1.0)	2.6 (2.2)	0.7 (0.4–1.0) units	**0.029**
POD1	1.4 (0.5)	1.7 (0.6)	0.3 (0.2–0.4) units	0.312
POD2	1.3 (0.5)	1.8 (0.6)	0.5 (0.4–0.6) units	**0.026**
POD3	1.4 (0.5)	1.7 (0.6)	0.3 (0.2–0.4) units	0.312

*Note:* Bolded values indicate statistical significance at a threshold of *p* < 0.05.

Abbreviations: CI, confidence interval; POD, postoperative day; pRBC, packed red blood cells; TXA, tranexamic acid.

^a^ *p*‐value compares patients who did versus did not receive TXA. Data presented as mean (SD), unless otherwise indicated. Relative risk considers no TXA as the reference group.

### 3.2 Intraoperative Transfusion

There was no difference in the proportion of patients requiring an intraoperative transfusion of pRBC between those who did and did not receive TXA (31% [52/166] vs. 33% [147/451]; *p* = 0.8) (Table 3). However, among patients who required transfusion, those who received TXA required significantly fewer intraoperative units of pRBC compared with patients who did not receive TXA (mean ± SD: 1.9 ± 1.0 units vs. 2.6 ± 2.2 units; *p* = 0.029) (Table 2).

**Table 3:** Perioperative transfusion rates.

	TXA (*n* = 166)	No TXA (*n* = 451)	Relative risk (95% CI)	*p*‐value^a^
Intraoperative transfusion rate	31% (52)	33% (147)	0.96 (0.74–1.25)	0.752
POD1 transfusion rate	5.4% (9)	12% (53)	0.46 (0.23–0.91)	**0.020**
POD2 transfusion rate	7.2% (12)	13% (58)	0.56 (0.31–1.02)	0.050
POD3 transfusion rate	7.2% (12)	11% (48)	0.68 (0.38–1.22)	0.202
Any transfusion POD1–POD3	17% (28)	30% (135)	0.56 (0.39–0.81)	**0.003**

*Note:* Bolded values indicate statistical significance at a threshold of *p* < 0.05.

Abbreviations: CI, confidence interval; POD, postoperative day; TXA, tranexamic acid.

^a^ *p*‐value compares patients who did versus did not receive TXA. Data presented as % (*n*), unless otherwise indicated. Relative risk considers no TXA as the reference group.

### 3.3 Postoperative Transfusion

Perioperative TXA administration was associated with a significantly lower risk of requiring a pRBC transfusion on postoperative day (POD) 1 compared with patients who did not receive TXA (5.4% [9/166] vs. 12% [53/451]; RR [95% CI]: 0.46 [0.23–0.91]; *p* = 0.020) (Table 3). However, among patients who required transfusion on POD1, there was no detectable difference in the number of transfused pRBC units between the TXA and no‐TXA groups (mean ± SD: 1.4 ± 0.5 units vs. 1.7 ± 0.6 units; *p* = 0.3) (Table 2).

TXA administration was also associated with a lower risk of requiring a postoperative pRBC transfusion on POD2 (7.2% [12/166] vs. 13% [58/451]; RR [95% CI]: 0.56 [0.31–1.02]; *p* = 0.050) (Table 3). In addition, patients who received TXA required significantly fewer total units of pRBC on POD2 compared with those who did not receive TXA (mean ± SD: 1.3 ± 0.5 units vs. 1.8 ± 0.6 units; *p* = 0.026) (Table 2).

On POD3, TXA administration was not associated with a significantly lower risk of requiring a postoperative pRBC transfusion (7.2% [12/166] vs. 11% [48/451]; RR [95% CI]: 0.68 [0.38–1.22]; *p* = 0.2) (Table 3), nor was there a significant difference in transfusion volume among transfused patients (mean ± SD: 1.4 ± 0.5 units vs. 1.7 ± 0.6 units; *p* = 0.3) (Table 2).

### 3.4 Length of Stay

Receiving perioperative TXA was associated with a significantly shorter postoperative hospital length of stay (mean ± SD: 4.9 ± 4.3 days vs. 5.7 ± 8.9 days; *p* = 0.003).

### 3.5 Complications

When comparing TXA‐treated and untreated patients, there was no statistically detectable difference in the risk of VTE complications within 90 days, including DVT, PE, MI, ischemic stroke/TIA, or other VTE (Table 4). Furthermore, there was no detectable difference in the rate of return to the operating room within 90 days between patients who did versus did not receive TXA (13% [22/166] vs. 15% [66/451]; RR [95% CI]: 0.91 [0.58–1.42]; *p* = 0.664). Finally, there was no detectable difference in the risk of 90‐day all‐cause mortality between patients who did vs did not receive TXA (11% [18/166] vs. 9.3% [42/451]; RR [95% CI]: 1.16 [0.69–1.96]; *p* = 0.569).

**Table 4:** Complications within 90 days of endoprosthetic reconstruction.

	All patients (*n* = 617)	TXA (*n* = 166)	No TXA (*n* = 451)	Relative risk (95% CI)	*p*‐value^a^
DVT	2.4% (15)	3.6% (6)	2.0% (9)	1.81 (0.65–5.01)	0.249
PE	2.1% (13)	3.6% (6)	1.6% (7)	2.33 (0.79–6.83)	0.123
MI	0.5% (3)	1.2% (2)	0.2% (1)	5.43 (0.50–59.5)	0.178
Ischemic stroke or TIA	0.3% (2)	0.6% (1)	0.2% (1)	2.72 (0.17–43.2)	0.457
Other VTE	1.0% (6)	0.6% (1)	1.1% (5)	0.54 (0.06–4.62)	0.999
Return to OR	14% (88)	13% (22)	15% (66)	0.91 (0.58–1.42)	0.656
All‐cause mortality	9.7% (60)	11% (18)	9.3% (42)	1.16 (0.69–1.96)	0.569

Abbreviations: CI, confidence interval; DVT, deep vein thrombosis; MI, myocardial infarction; OR, operating room; PE, pulmonary embolism; TIA, transient ischemic attack; TXA, tranexamic acid; VTE, venous thromboembolism.

^a^ *p*‐value compares patients who did versus did not receive TXA. Data presented as % (*n*), unless otherwise indicated. Relative risk considers no TXA as the reference group.

## 4 Discussion

While TXA has become broadly used across many orthopedic subspecialties, its use within orthopedic oncology remains relatively controversial. Previous studies exploring the use of TXA in orthopedic oncology populations have revealed contradictory findings with respect to effects on blood loss and rates of VTE complications. In a large cohort of patients undergoing lower extremity endoprosthetic reconstruction for oncologic indications, we found that patients who received TXA had significantly lower blood loss, required fewer transfusions, and had no detectable increase in thromboembolic complications compared to patients who did not receive TXA.

These results are generally well‐aligned with previous studies of TXA in orthopedic oncology populations. The strongest available evidence to date comes from a single‐center, randomized, double‐blinded, placebo‐controlled trial of 48 pediatric patients undergoing endoprosthetic reconstruction for malignant bone tumors of the femur. In this study, the 24 patients who received TXA had significantly lower perioperative blood loss and transfusion requirements with no differences in rates of PE or deep venous thrombosis [36].

Multiple small, retrospective studies have reported similar results. In a cohort of 90 patients undergoing endoprosthetic prosthetic reconstruction for oncologic indications, the 34 patients who received TXA had significantly lower perioperative blood loss, decreased transfusion rate, decreased transfusion volume, reduced hospital length of stay, and no difference in VTE rate [29]. In a study of 56 pediatric patients undergoing distal femoral endoprosthetic reconstruction for osteosarcoma, the 25 patients who received TXA had significantly lower intraoperative blood loss, lower postoperative drain output, lower transfusion rate, shorter hospitalization, and no difference in VTE events [30]. Comparable findings were observed in a study of 61 patients undergoing endoprosthetic reconstruction for primary or metastatic bone tumors, where the 30 patients who received TXA had significantly lower blood loss, lower transfusion volume, and no difference in VTE rate [31]. In another retrospective study of 40 adult patients who underwent endoprosthetic reconstruction following sarcoma resection, the 22 patients who received TXA had significantly lower intraoperative and postoperative transfusion volumes and shorter operative time, with no difference in VTE rate [34].

In a secondary analysis of the PARITY trial, it was found that the use of intraoperative TXA was not significantly associated with the occurrence of a thromboembolic event, nor was its use associated with reoperation for irrigation and debridement [43]. In a retrospective study of 4497 patients undergoing a variety of surgical interventions for neoplastic pathologic fractures of the lower extremity, the 769 patients who received TXA had significantly *lower* rates of DVT, MI, acute blood loss anemia, and 90‐day in‐hospital death [35].

In contrast, a study by Foster et al. is the only report to our knowledge describing a positive association between TXA use and the development of PE in patients with musculoskeletal sarcomas [37]. However, the statistical approach used in this study limits the validity of its conclusions. The authors employed stepwise backward selection to construct a multivariable model despite having only 26 PE events, a methodology that is well recognized to produce biased coefficient estimates, exaggerated effect sizes, and unreliable p‐values, particularly in small datasets [44]. Given the limited number of events, the final model included only two variables, TXA use and DVT, thereby excluding multiple clinically relevant confounders such as tumor size, anatomic location, grade, stage, and thromboprophylaxis regimen, all of which differed at baseline between treatment groups. Moreover, inclusion of DVT as a covariate when modeling PE is problematic, as these outcomes are closely related and occur within the same 90‐day postoperative period, making DVT an inappropriate predictor of PE in this context. Most importantly, the use of stepwise selection rather than a priori specification of confounders results in TXA serving as a surrogate marker for more extensive, anatomically complex, and biologically aggressive tumors, which are independently associated with higher thrombotic risk. As a result, the reported association between TXA use and PE in this study cannot be reliably interpreted as causal.

A robust meta‐analysis examining TXA use in sarcoma surgery was published by Giglio et al. the following year [27]. This meta‐analysis included eight studies comprising 2142 patients with sarcoma and demonstrated no difference in VTE risk between patients who did and did not receive TXA (OR = 0.93 [95% Cl: 0.40, 2.16]). Notably, the only study within the meta‐analysis that identified TXA as a risk factor for any type of VTE was the report by Foster et al, discussed above [37]. The authors of the meta‐analysis also noted the findings of the study by Foster et al. were “likely secondary to selection bias given that patients with larger, pelvic‐based tumors had higher rates of TXA utilization” [27].

It is important to recognize that the antifibrinolytic mechanism of TXA does not equate to the induction of a hypercoagulable state, nor does it necessarily exacerbate the baseline hypercoagulability associated with malignancy. Furthermore, experimental data demonstrate that neither plasminogen activation nor plasmin activity is required for protection against venous thrombosis or venous thromboembolism [45]. Accordingly, when considering TXA use in high‐risk oncologic patients, the well‐established benefits of reduced blood loss must be weighed against an increased thromboembolic risk that remains largely unsubstantiated.

Finally, TXA may confer additional benefits that are currently underrecognized or incompletely understood. Beyond its hemostatic effects, TXA has been hypothesized to exert anticancer activity through modulation of tumor cell proliferation, angiogenesis, inflammation, and tissue remodeling. In a recent meta‐analysis encompassing 41 in vitro studies, 34 animal studies, and 7 clinical studies involving 91 patients, TXA‐treated groups demonstrated reduced tumor growth compared with controls [46]. Although preliminary, these findings support further investigation into the mechanisms and potential magnitude of any antineoplastic effect of TXA in orthopedic oncology patient populations.

## 5 Limitations

This work is subject to all the biases and constraints inherent to its single‐center, retrospective study design. Selection bias is likely present in the decision to administer TXA, consistent with prior studies in this domain. However, the issue of selection bias warrants careful interpretation. Patients with larger and more anatomically complex tumors typically undergo longer operations and experience prolonged postoperative immobility, which are factors that independently increase the risk of DVT and PE. From a hemostatic standpoint, however, these same patients are also the most likely to benefit from TXA, as extensive oncologic resections are associated with the greatest blood loss and transfusion requirements.

**Figure 2:**
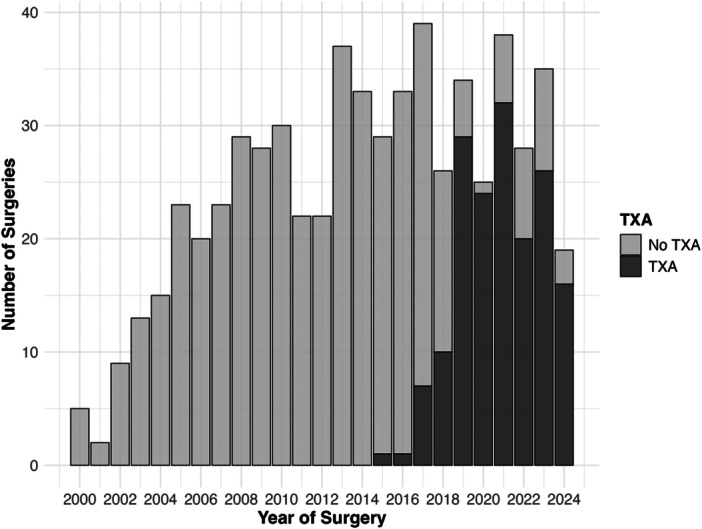
Histogram demonstrating the use of tranexamic acid (TXA) for lower extremity oncologic endoprosthesis surgery over time.

In addition, the year of surgery represents an important confounder, as TXA use increased substantially over the study period (Figure 2). Consequently, the non‐TXA cohort effectively represents a phased historical comparison rather than a contemporaneous control group. Therefore, the observed reduction in length of stay among patients who received TXA is likely confounded by changes in hospital practices and administrative policies that prioritize earlier discharge, limiting the ability to infer a direct relationship between TXA administration and hospital length of stay. Similarly, just as surgical technique and implant preferences have changed over time, perioperative transfusion practices likely changed over the course of the study period as orthopedic oncology has moved towards more restrictive transfusion triggers. Despite this limitation, the decrease in calculated perioperative blood loss in the TXA group corresponded with decreased transfusion rates.

Furthermore, postoperative VTE chemoprophylaxis was not included in the analysis. Although this information was abstracted from the electronic medical record, documentation was missing or incomplete for more than 40% of patients. Even when chemoprophylaxis was recorded, reliable assessment of patient adherence was not possible. Interpretation is further complicated by variability in provider practice patterns and temporal changes in prescribed regimens, including transitions from low‐molecular‐weight heparin to aspirin and the increasing use of direct factor Xa inhibitors in place of warfarin.

Finally, thromboembolic complications are rare events, with previously literature reporting the rate of PE in this population to be approximately 1.6% [47]. Based on this reference standard, the present study is only powered to detect a 5.7% absolute difference in PE rate between groups. Therefore, this study is underpowered to detect small differences in VTE risk should they truly exist, raising the possibility of a type II error. To detect a true 2% absolute difference between groups, over 2000 patients would be required. Accordingly, multicenter prospective studies will be necessary to address these limitations and to provide high‐quality evidence to guide TXA use in this high‐risk orthopedic oncology population.

## 6 Conclusions

In this single‐center study of 617 patients undergoing lower extremity endoprosthetic reconstruction for oncologic indications, perioperative TXA use was associated with significantly reduced blood loss and transfusion requirements, without a detectable increase in thromboembolic complications. These findings support TXA as a beneficial adjunct that may be used with appropriate caution in patients with musculoskeletal malignancies undergoing large limb salvage procedures. Future prospective studies and multicenter collaborations are warranted to provide higher‐quality evidence to further define the safety and optimal use of TXA in this high‐risk orthopedic oncology population.

## Conflicts of Interest

Research reported in this publication was supported by NIGMS of the National Institutes of Health under award numbers T32GM007347 and T32GM152284. Additional support included the training grant T32AR085529 from the National Institute of Arthritis and Musculoskeletal and Skin Diseases. Each author certifies that there are no funding or commercial associations (consultancies, stock ownership, equity interest, patent/licensing arrangements, etc.) that might pose a conflict of interest in connection with the submitted article related to the author or any immediate family members.

## Synopsis

Tranexamic acid (TXA) adoption in orthopedic oncology has been limited by concerns about thromboembolic risk in patients with cancer. In this retrospective study of 617 patients undergoing lower extremity endoprosthetic reconstruction for oncologic indications, TXA reduced perioperative blood loss by 429 mL and postoperative transfusion requirements without detectable increases in 90‐day venous thromboembolic complications, reoperation rates, or mortality. These findings support the use of TXA as a beneficial adjunct in musculoskeletal oncology limb salvage procedures.

## Data Availability

Data sharing not applicable to this article as no datasets were generated or analyzed during the current study.
